# Downregulation of the Vitamin D Receptor Regulated Gene Set in the Hippocampus After MDMA Treatment

**DOI:** 10.3389/fphar.2018.01373

**Published:** 2018-12-03

**Authors:** Peter Petschner, Noemi Balogh, Csaba Adori, Viola Tamasi, Sahel Kumar, Gabriella Juhasz, Gyorgy Bagdy

**Affiliations:** ^1^Department of Pharmacodynamics, Semmelweis University, Budapest, Hungary; ^2^MTA-SE Neuropsychopharmacology and Neurochemistry Research Group, Hungarian Academy of Sciences, Semmelweis University, Budapest, Hungary; ^3^Department of Genetics, Cell- and Immunobiology, Semmelweis University, Budapest, Hungary; ^4^SE-NAP 2 Genetic Brain Imaging Migraine Research Group, Hungarian Brain Research Program, Semmelweis University, Budapest, Hungary; ^5^NAP-2-SE New Antidepressant Target Research Group, Hungarian Brain Research Program, Semmelweis University, Budapest, Hungary

**Keywords:** vitamin D, ecstasy, long-term potentiation, gene expression, microarray, hippocampus

## Abstract

The active ingredient of ecstasy, ±3,4-methylenedioxymethamphetamine (MDMA), in addition to its initial reinforcing effects, induces selective and non-selective brain damage. Evidences suggest that the hippocampus (HC), a central region for cognition, may be especially vulnerable to impairments on the long-run, nevertheless, transcription factors that may precede and regulate such chronic changes remained uninvestigated in this region. In the current study, we used gene-set enrichment analysis (GSEA) to reveal possible transcription factor candidates responsible for enhanced vulnerability of HC after MDMA administration. Dark Agouti rats were intraperitoneally injected with saline or 15 mg/kg MDMA. Three weeks later HC gene expression was measured by Illumina whole-genome beadarrays and GSEA was performed with MSigDB transcription factor sets. The number of significantly altered genes on the genome level (significance < 0.001) in up/downregulated sets was also counted. MDMA upregulated one, and downregulated 13 gene sets in the HC of rats, compared to controls, including Pax4, Pitx2, FoxJ2, FoxO1, Oct1, Sp3, AP3, FoxO4, and vitamin D receptor (VDR)-regulated sets (*q*-value <0.05). VDR-regulated set contained the second highest number of significantly altered genes, including among others, Camk2n2, Gria3, and Grin2a. Most identified transcription factors are implicated in the response to ischemia confirming that serious hypoxia/ischemia occurs in the HC after MDMA administration, which may contribute to the selective vulnerability of this brain region. Moreover, our results also raise the possibility that vitamin D supplementation, in addition to the commonly used antioxidants, could be a potential alternative method to attenuate MDMA-induced chronic hippocampal impairments.

## Introduction

Ecstasy is a commonly abused drug worldwide for its stimulating, pro-social and euphoric effects (Green et al., 2003). The active ingredient of ecstasy is 3,4-methylenedioximethamphetamine (MDMA) that acutely releases noradrenaline, serotonin, and dopamine in synaptic clefts (Green et al., 2003; Pazmany et al., 2013). Besides of its initial rewarding effects, the drug can lead to lasting functional consequences: elevated risk of mood disorders and persistent cognitive disturbances were demonstrated in multiple animal and human studies (Parrott et al., 2000; Green et al., 2003; Bond et al., 2004; Kalechstein et al., 2007; Nulsen et al., 2010). A central region of cognitive functions, particularly memory, is the hippocampus (HC) (Sweatt, 2004). Its anatomical structure, in terms of organization of neuronal circuitries and (micro)vasculature makes it especially vulnerable to various homeostatic challenges (Schmidt-Kastner and Freund, 1991). The HC seems to be the most vulnerable brain region also to the toxic effects of MDMA in rats and, with some suggestive evidence, in humans (Adori et al., 2006, 2010; den Hollander et al., 2012; Petschner et al., 2018). MDMA-induced hyperthermia, free radical production, relative ischemia through disruption of cerebral autoregulation (Colado et al., 1999; Green et al., 2003; Ferrington et al., 2006; Capela et al., 2009) and lasting selective serotonergic toxicity (Kirilly et al., 2006; Capela et al., 2009; Adori et al., 2010, 2011b; Ando et al., 2010) are all mechanisms that could contribute to the damage of HC. Translation of these complex pathophysiological processes to molecular mechanisms leading to the damage of the HC and to impairments of learning and memory processes are, however, less explored, although changes in LTP in hippocampal slices after neurotoxic MDMA treatment have been described earlier (Morini et al., 2011). We have demonstrated previously that mRNA levels of key members of the long-term potentiation (LTP) pathway were downregulated in the HC 3-weeks after MDMA administration, in parallel with decreased expression of memory and cognition gene sets (Petschner et al., 2018). These may be important contributors to MDMA-induced cognitive impairments. However, based on the complexity of the pathophysiological processes mentioned above, further molecular mechanisms are presumed to contribute. These probably employ downstream mediators, like transcription factors, which regulate expression of mRNA and protein levels directly, and link initial alterations with long-term consequences. Nevertheless, transcription factors remained uninvestigated after chronic MDMA administration.

In the present study, we have analyzed our previously obtained data, measured by Illumina whole-genome beadarrays, with gene-set enrichment analysis (GSEA) using the MSigDB transcription factor sets, to identify transcription factors that may be responsible for the long-term cognitive impairments in the vulnerable HC region 3-weeks after a single-dose MDMA administration.

## Materials and Methods

For detailed methods see (Petschner et al., 2018). Briefly, 21 approximately 8 weeks old male Dark Agouti (DA) rats (Harlan, Olac Ltd., Shaw’s Farm, Blackthorn, Bicester, United Kingdom) weighing 152 ± 3,58 g (SEM) were used. After random assignment, MDMA [(±)3,4-methylenedioxymethamphetamine (Sanofi-Synthelabo-Chinoin, Hungary, purity >99.5%)] dissolved in 0.9% NaCl (SAL), at an equivalent dose of 15 mg/kg free base, or SAL (for control animals) was administered intraperitoneally in a volume of 1 ml/kg. Three weeks after injections rats were sacrificed and HC regions dissected (dorsal HC: from bregma -2.5 to -4.5 mm). RNA extraction was performed according to the TRIzol method (Tamasi et al., 2014; Petschner et al., 2016). Samples with lowest RNA concentrations were excluded until both MDMA and control groups consisted of eight animals. Randomly selected samples were pooled by two and sent to Service XS (Leiden, Netherlands) for microarray analysis with the Illumina (San Diego, CA, United States) RatRef-12v1 beadarray expression chip. Raw data were analyzed using quantile normalization with beadarray, preprocessCore and puma Bioconductor packages for R (Gentleman et al., 2004; Dunning et al., 2007; Pearson et al., 2009; R Core Team, 2012). These methods created the minimum probability of positive log ratio (MinPplr), a statistical significance value. A gene was considered significant, if MinPplr was below 0.001 [to correct for multiple hypothesis testing (Benjamini and Hochberg, 1995; Petschner et al., 2015)]. Results were validated with rt-PCR (for result see Petschner et al., 2018) and microarray data uploaded in Gene Expression Omnibus (Edgar et al., 2002) under GSE47541^1^. GSEA was performed using GSEA software from the Broad Institute at MIT^2^. GSEA uses a ranked list of all examined genes and searches for groups of genes (gene sets) that are enriched (overrepresented/underrepresented) in the top/bottom of this list. We used official MSigDB C3 TFT (transcription factor) gene sets for grouping, which classifies genes based on shared regulatory motifs and, hence, represents common transcription factor binding properties. Accordingly, up/downregulation of a given set in GSEA means that genes regulated by a given factor are up/downregulated. Sets containing more than 500/less than 15 genes were excluded, *t*-test was used for ranking and gene set was chosen as permutation type. Thousand permutations were made with seed of permutation set to 149. Normalized enrichment scores (NES), representing relative enrichment in a given phenotype, nominal *p*-values and false discovery rates (FDR) were calculated. A gene set was considered significant with FDR *q*-value below 0.05 (Benjamini and Hochberg, 1995). GSEA calculates results obtained through analysis of every (both significant/non-significant) genes between two groups (in this case MDMA vs. SAL). However, we were also interested in sets that may contain most of the significantly altered genes. Therefore, the number of significantly altered genes (MinPplr < 0.001) was counted in each significant (FDR < 0.05) gene set to identify those transcription factors that may be largest contributors to the most substantial changes in the HC 3 weeks after administration.

All experiments complied with the European Community Council Directive of 24 November 1986 (86/609/EEC), National Institutes of Health Principles of Laboratory Animal Care (NIH Publication 85-23, revised 1985) and national laws (the Hungarian Governmental Regulation on animal studies, 31 December 1998 Act) and were approved by the National Scientific Ethical Committee on Animal Experimentation and permitted by the Food Chain Safety and Animal Health Directorate of the Central Agricultural Office, Hungary (permission number: 22.1/3152/001/2007).

## Results

Gene-set enrichment analysis revealed 14 gene sets that were dysregulated with an FDR *q*-value below 0.05 3 weeks after a single-dose MDMA treatment in the HC compared to the controls (Table 1). Among the gene sets one was upregulated, the other 13 were downregulated with the largest downregulation of the Pax4 set (the only upregulated set possesses no known binding factor). From the 14 dysregulated gene sets three bind forkhead box (Fox) transcription factors, namely FoxO1, FoxO4, and FoxJ1, others could not be grouped into families.

**Table 1:** Up- and downregulated transcription factor gene sets 3 weeks after a single-dose MDMA treatment in the hippocampus of Dark Agouti rats.

Name of the gene set	Presumed transcription factor able to bind to regulatory motif	NES	FDR *q*-value
ACTWSNACTNY_UNKNOWN	Unknown	1.936839	0.022079093
V$VDR_Q6	Vitamin D receptor	-1.7801763	0.047251813
AACWWCAANK_UNKNOWN	Unknown	-1.8135455	0.03694073
V$FOXO4_01	FoxO4	-1.8182034	0.038588446
GTGGGTGK_UNKNOWN	Unknown	-1.8234655	0.039833214
V$AP3_Q6	Currently unknown	-1.8274099	0.042258345
V$SP3_Q3	Sp3	-1.8449765	0.03698938
V$COREBINDINGFACTOR_Q6	Core-binding factor	-1.8531265	0.038751207
V$OCT1_03	Oct1 (Pou2f1)	-1.8553681	0.04364336
V$FOXO1_01	FoxO1	-1.8712838	0.042675186
V$FOXJ2_02	FoxJ2	-1.8762817	0.049202178
YRCCAKNNGNCGC_UNKNOWN	Unknown	-1.9217957	0.037266485
V$PITX2_Q2	Pitx2	-1.9697808	0.025443465
V$PAX4_04	Pax4	-2.1240275	0.0034508866


*According to the gene set enrichment analysis, altogether 14 gene sets were dysregulated (1 up- and 13 downregulated, marked with red and blue, respectively) with a false discovery rate (FDR) below 0.05. All of the significant sets are shown here in descending order, according to their nominal enrichment scores (NES), thus, toward lower expression of their respective genes. Table contains the most probable transcription factors that may bind regulatory motifs. Those genes, that were annotated to a set based only on common regulatory motifs, but unknown transcription factors are marked as “unknown” in their respective columns (four sets). AP3 set currently lacks associated transcription factor.*

To reveal gene sets with the highest number of altered genes, we counted significant gene entries (MinPplr < 0.001) in each significant set (FDR < 0.05). Results showed that AP3 regulated gene set contained the most significantly altered genes with 17 entries, [but AP3 is currently not associated with known transcription factor (see section “Discussion”)], followed by vitamin D receptor (VDR) regulated set with 15 entries. Results with the significant genes are shown in Table 2.

**Table 2:** Number of genes with significantly altered expression 3 weeks after MDMA treatment in the hippocampus in the significantly altered transcription factor gene sets.

Gene set	Number of sign. Genes							Genes
V$AP3_Q6	17	PPM1E	CRK	SEPHS2	ADCY8	DDX6	PLAG1	TRPS1	CDH8	SCA2	IGF2	NTRK2	CDH9	MPP6	CXXC4	SLC5A3	RORA	NTRK3
V$VDR_Q6	15	CEBPB	CD3E	CAMK2N2	KLF1	PMP22	PABPC1	ATP2B3	GRIA3	SLC4A4	NTRK2	RBBP6	LPHN1	DNMT3A	TNR	GRIN2A		
V$PITX2_Q2	12	DCTN4	SEMA5B	LPHN2	TRPS1	LRP6	PRICKLE2	NTRK2	DP1	GSK3B	RORA	BMPR2	NFIX					
V$SP3_Q3	12	DNAJB5	BAI2	KLF1	SH3BP5	PABPC1	KDELR1	PLAG1	ENH	ASCL1	DNMT3A	GSK3B	GRIN2A					
V$PAX4_04	11	RFX3	UBE2B	PLAG1	GRIA3	NTRK2	TCF4	GNAO	GSK3B	SLC5A3	TNR	NFIX						
V$FOXO4_01	11	KLF1	ZHX2	SH3BP5	ITGA1	LPHN2	PLAG1	TRPS1	SLC4A4	ENPP2	LPHN1	NTRK3						
V$FOXO1_01	11	CAMK2N2	KLF1	SH3BP5	IGSF1	LPHN2	PLAG1	PLXDC2	POU3F2	MGLL	NTRK3	NFIX						
GTGGGTGK_UN-KNOWN	11	NCK2	KLF1	ZHX2	ACVR1C	ATP2B3	MYCL1	BASP1	IGF2	DLGAP4	KCND2	CDH22						
V$OCT1_03	9	KLF1	PRKG1	AMD1	PLAG1	TCF4	DLGAP4	GDA	CDH22	NFIX								
V$FOXJ2_02	9	GNAZ	ADAM23	ACVR1C	NFIA	CDH9	TCF4	RORA	BMPR2	NFIX								
AACWWCAANK_UNKNOWN	8	RCN3	RPS6KA5	LPHN2	PLAG1	IGF2	ALCAM	PCDH17	BMPR2									
V$COREBINDING-FACTOR_Q6	6	MAFG	ENPP1	ENPP2	GSK3B	BMPR2	NFIX											
ACTWSNACTNY_UNKNOWN	5	SLC9A3R1	NRAP	RCN3	TRPS1	MPP6												
YRCCAKNNGNCGC_UNKNOWN	3	BAI2	MYCL1	GRIN2A														


*The table enlists names of genes that were significantly up/downregulated (MinPplr < 0.001) in the gene expression analysis and belonged to one of the significantly altered transcription factor gene sets 3 weeks after single-dose MDMA treatment in rat hippocampi. Results show that AP3 transcription factor regulated genes are most prominent while vitamin D receptor gene set also contained 15 genes from the significantly altered ones. Red genes represent those that were significantly (MinPplr < 0.001) upregulated, while blue ones were downregulated. Genes are represented by their respective official symbols.*

## Discussion

The present study shows that gene expression alterations 3 weeks after a single-dose MDMA treatment in the HC are probably mediated by 14 different transcription factors, among them hypoxia/ischemia-induced members of the FoxO family, Sp3 and Pax4. The AP3 regulated set contained the most significantly dysregulated genes, but lacks associated transcription factor, while the VDR gene set contained the second most entries. These results suggest that etiologically hypoxia/ischemia may be an important factor in the vulnerability of the HC and vitamin D supplementation may be tested to compensate for the most substantial cognitive effects of the drug.

Among transcription factors regulating the significantly altered sets several could be related to hypoxia/ischemia, a known consequence of MDMA administration (Ferrington et al., 2006). For example, the Pax4 regulated gene set was downregulated after transient hypoxic condition in the fetal pituitary of sheep (Wood et al., 2016). FoxO1 and FoxO4 were implicated in antioxidant mechanisms after brain ischemia (Abbas et al., 2009; Fukunaga and Shioda, 2009; Araujo et al., 2011; Jenwitheesuk et al., 2017). Polymorphisms in Pitx2 associated consistently with ischemic stroke in genome-wide association studies [Traylor et al., 2012; Malik et al., 2016; Ninds Stroke Genetics Network (SiGN) and International Stroke Genetics Consortium (Isgc), 2016]. Significantly decreased Sp3 protein levels were revealed after 7 days of hypobaric hypoxia in HC of rats (Hota et al., 2010). Other transcription factors had wide-scale roles in different processes, e.g., FoxJ2 is an important regulator of dopaminergic neurons (Wijchers et al., 2006) and Oct1 (Pou2f1) polymorphisms associated with Alzheimer’s disease (Taguchi et al., 2005). Core-binding factor is a large complex from Runx1-3 and core-binding factor B, which possesses important transcriptional regulatory properties, and Runx1 was induced after traumatic brain injury (Logan et al., 2013). From the dysregulated gene sets in four, including the only upregulated one, genes are annotated only by common regulatory motifs that are not associated with known transcription factors, indicating that so far unidentified regulatory elements may also contribute to the long-term effects of MDMA. In sum, our results suggest that MDMA employs FoxO1/4, Pitx2, Sp3, and Pax4-dependent mechanisms after its initial vasoactive monoamine release and consequent ischemia/hypoxia and changes in these transcription factors may be responsible for the long-lasting influence on HC processes and the often observed cognitive impairments.

From this aspect, perhaps the most interesting result is that most of the VDR regulated genes were downregulated (see Table 2). In fact, the second highest number of genes with significantly altered expressions were among the VDR regulated genes, including those involved in LTP, like upregulated calcium/calmodulin-dependent kinase inhibitor *Camk2n2* and downregulated glutamate receptors NMDA2A (*Grin2a*) and AMPA3 (*Gria3*). While some of the other transcriptional regulators may also influence genes important for regulation of LTP, none of them offer such straightforward intervention as VDR regulated one in the form of vitamin D supplementation. This is especially tempting for multiple reasons. First, vitamin D exerts its effects via VDR, which after activation binds directly to DNA and influences gene transcription (Whitfield et al., 1995; Haussler et al., 1997; Angelo et al., 2008). Vitamin D was demonstrated to counteract consequences of hypoxia/ischemia via VDR (Won et al., 2015), showed neuroprotective effects in hippocampal neurons after hypoxic insult (Kajta et al., 2009), could influence LTP (Salami et al., 2012), enhanced cognitive performance in dementia (Landel et al., 2016), and protected from the serotonin-depleting effects of methamphetamine, a close relative of MDMA (Cass et al., 2006). Furthermore, a transient decrease in plasma calcitriol level was demonstrated in humans by a metabolomic analysis after acute MDMA administration (Boxler et al., 2018) and ecstasy users promote the use of vitamin D in user-to-user posts^3^. However, no previous studies investigated vitamin D effects after MDMA use (in PubMed no hit with “MDMA” and “vitamin D” MeSH terms, retrieved on 11th of August, 2018). Harm-reduction techniques, found overall on the internet, target known acute actions of MDMA and suggest application of antioxidants, hydration, and cooling to attenuate the negative effects. Among those cooling was extensively studied and showed limited success (see e.g., Adori et al., 2011a). Nevertheless, based on our results, vitamin D might have the potential to compensate for some of the long-term consequences of MDMA in the highly vulnerable HC.

## Conclusion

While our study has to be interpreted with caution due to the indirect examination of the transcription factors and the exclusive investigation of the HC, it provides evidence for an important role of ischemia and subsequent FoxO1/4, Pitx2, Sp3, and Pax4-dependent mechanisms in MDMA-induced hippocampal impairments. In addition, it also raises the possibility of a better, simple, and precision-medicine based harm reduction technique with vitamin D to compensate for the cognitive deficits of the drug.

## Author Contributions

PP conducted the GSEA and gene level analysis, interpreted the data, and wrote and drafted the manuscript. NB helped in the GSEA analysis. CA carried out the treatment protocol, dissected the brain samples, and prepared the RNA extractions. VT and SK participated in the interpretation and contributed in drafting the manuscript. GJ helped to draft the manuscript. GB prepared the study design, coordinated the study, and helped in the interpretation and drafting the manuscript. All authors have read and approved the final manuscript.

## Conflict of Interest Statement

The authors declare that the research was conducted in the absence of any commercial or financial relationships that could be construed as a potential conflict of interest.

## Footnotes

1 http://www.ncbi.nlm.nih.gov/geo/query/acc.cgi?acc=GSE47541

2 http://www.broadinstitute.org/gsea

3 www.treato.com
